# Editorial: *O*-GlcNAcylation: Expanding the Frontiers

**DOI:** 10.3389/fendo.2019.00867

**Published:** 2019-12-13

**Authors:** Tarik Issad, Tony Lefebvre

**Affiliations:** ^1^Université de Paris, CNRS UMR8104, INSERM U1016, Institut Cochin, Paris, France; ^2^Université de Lille, CNRS, UMR 8576, UGSF, Lille, France

**Keywords:** *O*-linked N-acetylglucosaminylation, post-translational modification, epigenetics, inflammation, cardiovascular diseases, cancer, *O*-GlcNAcomics

*O*-linked N-acetylglucosaminylation (*O*-GlcNAcylation) is a fascinating post-translational modification (PTM) that controls numerous biological processes, including epigenetic regulations, gene expression, proteostasis, energy metabolism, cell signaling, growth and proliferation (1). Whereas it essentially remained confidential during the first decades following its discovery, the importance of this protein modification in life sciences is now undisputed. In 2014, we celebrated the 30 years anniversary of its discovery by editing a Research Topic in Frontiers in Endocrinology (2). As everyone can notice, time is speeding up and this is particularly true in Science. Almost 5 years now after launching the publication of this Research Topic, it seems to us that significant progresses and new discoveries have been made in the field that justify another issue on this subject.

Alike phosphorylation, *O*-GlcNAcylation is a reversible PTM that occurs on serine and threonine residues of cytosolic, nuclear and mitochondrial proteins. Only two enzymes, OGT (*O*-GlcNAc transferase) and OGA (*O*-GlcNAcase), control the *O*-GlcNAc dynamics on proteins. OGT utilizes UDP-GlcNAc, provided by the hexosamine biosynthesis pathway (HBP), to add GlcNAc on proteins, whereas OGA removes it. UDP-GlcNAc, which is at the cross-road of several metabolic pathways, is considered as a sensor of the nutritional state of the cell. Thus, *O*-GlcNAcylation regulates cellular homeostasis according to metabolic environment, but also in response to signaling molecules (e.g., hormones and cytokines). Widespread in most living organisms studied so far, *O*-GlcNAcylation is highly susceptible to stress and injuries, and is involved in many human pathologies including diabetes, cancer and neurodegenerative diseases (3–5).

This E-book, which comprises reviews and original articles, provides up-to date examples of the implication of *O*-GlcNAcylation in diverse organisms and of its role in various physiological and pathological processes.

A remarkable feature of *O*-GlcNAcylation is its capacity to modulate other PTMs, which confers it the capability to interfere with various signaling and nutrient sensing pathways. AMP-activated protein kinase and mTOR pathways, regulated by AMP/ATP ratio and amino-acid levels, respectively, are major players in nutrient sensing. Reviews by Gélinas et al., Cork et al., and Very et al. extensively discuss different aspects of the dialogues between HBP, OGT, AMPK, and mTOR signaling at the cellular and molecular levels, as well as their dysregulation in pathophysiological situations. In the same vein, Ong et al. speculate on *O*-GlcNAc as an integrator of signaling pathways, emphasizing the necessity of maintaining what these authors called the “*O*-GlcNAc meter” at an optimal level, between a lower limit necessary for maintenance of cell structural integrity and critical functions, and a higher limit above which persistent *O*-GlcNAcylation leads to aberrant signaling.

Also, *O*-GlcNAcylation now appears as a major player in the immune system, and its role in inflammation constitutes an important area of investigation (6). In this Research Topic, two original articles deal with these aspects. Krick et al. provided data indicating that in human bronchial epithelial cells, FGF23, an important endocrine pro-inflammatory mediator, induces IL6 production. This effect is mediated by a FGFR4/Phospholipase Cγ/Nuclear factor activated T-cells (NFAT) signaling pathway and involves increased protein *O*-GlcNAcylation upon FGF23 stimulation. This work points out the potential role for *O*-GlcNAcylation in pathogenesis of chronic inflammatory airways diseases.

Chronic inflammation is also a hallmark of metabolic diseases such as diabetes and obesity. Hyperglycaemia is considered to be an important player in the initiation and persistence of inflammation associated with these pathologies (7). In pancreatic β-cells, high glucose concentrations stimulate the interaction of thioredoxin interacting protein (TxNIP) with the inflammasome protein NLRP3 (NLR family, pyrin domain containing 3), thereby promoting interleukin-1β (IL1β) maturation and secretion. Filhoulaud et al. demonstrated that TxNIP protein is modified by *O*-GlcNAcylation in rodent and human pancreatic β-cells, resulting in increased association with NLRP3, inflammasome activation, and production of mature IL1β. These data provide a new link between *O*-GlcNAcylation, inflammation, and glucotoxicity in pancreatic β-cells.

*O*-GlcNAcylation has largely been involved in diabetic complications associated with glucotoxicity, including cardiovascular dysfunctions (4). In the article by Mercier et al., a potential interplay between phosphorylation and *O*-GlcNAcylation of sarcomeric proteins in ischemic heart failure was examined, and the authors paid a particular attention to the intermediate filament structure essential component desmin. In addition, whereas the review by Ducheix et al. focused on the cellular and molecular mechanisms by which chronic *O*-GlcNAcylation affects cardiac function in diabetic cardiomyopathy, Ferron et al. evaluated the potential impact of modulating *O*-GlcNAc levels in acute cardiovascular pathologies, including haemorrhagic shock and myocardial ischemia-reperfusion injury.

The beneficial effects of physical exercise for prevention and treatment of human chronic diseases, including diabetes, cancer, and neurodegenerative diseases, are now largely documented. Skeletal muscle is quantitatively the most important glucose consumer tissue of the organism, and as such a crucial determinant of whole-body insulin sensitivity. It is also a major site of lipid oxidation and protein turn-over. In this Research Topic, Lambert et al. reviewed the involvement of *O*-GlcNAcylation in skeletal muscle chronic and acute exercise, as well as in pathophysiological situations, such as muscular atrophy or insulin resistance.

*O*-GlcNAcylation has also been involved in the control of cell cycle (8). One of the functions of the cell cycle is to replicate the DNA so that each daughter cell resulting from the division can inherit a copy conforming to the mother cell. But when the cell cycle is upset, errors in DNA replication can arise. *O*-GlcNAcylation intervenes both in the fine-tune control of cell cycle and in the maintenance of genome integrity by controlling DDR (DNA damage response). This field of particular interest is the topic of the review by Liu and Li who gave a comprehensive overview of the issue.

Cyclins are master regulators of the cell cycle. Cyclin D1 interacts with CDK4/6 to control cell cycle entry and progression in G1 phase. Masclef et al. showed that the fate of cyclin D1 depends upon *O*-GlcNAc status. Mechanistically, these authors demonstrated that cyclin D1 interacts with OGT; this leads to its *O*-GlcNAcylation which thwarts its ubiquitination and its subsequent targeting to the proteasome.

In this special issue, the role of *O*-GlcNAcylation in stemness was also investigated. Fuentes-García et al. showed that inhibition of OGT in colon cell lines interferes with the expression of the two stem cell markers CD44 and CD133, and coincides with an increased clonogenicity and spheroid formation capabilities. Of particular interest, the authors suggest that *O*-GlcNAc serves as a sensor giving the cancer cells the ability to face nutrient stressful conditions.

Dysregulation of *O*-GlcNAcylation is also associated with neurodegenerative disorders including Alzheimer's disease as previously documented (9). While a reciprocal relationship between *O*-GlcNAcylation and phosphorylation on Tau protein has been reported (10, 11), molecular details of this interaction remain largely unknown. Bourré et al. used NMR spectroscopy approach to map *O*-GlcNAc sites on the longest isoform of Tau and to gain insight into the crosstalk between *O*-GlcNAcylation and phosphorylation. They propose that both PTMs can affect Tau in a more intricate relationship than a single direct reciprocal manner. This interesting paper reinforces a little more the complexity of protein regulation by PTMs.

*O*-GlcNAcylation is now recognized as an important player in epigenetic regulations (12). In a mini-review, Decourcelle et al. compared the structure of the human and drosophila Polycomb Repressor Complexes and discuss their regulation by *O*-GlcNAcylation in drosophila embryonic development and in human cancer cells. Moreover, original work by Krause et al. evaluated the link between *O*-GlcNAcylation, nutritional status and epigenetic regulation of gene expression by studying genome wide RNA Polymerase II binding in response to starvation and feeding, in *C. elegans* mutants lacking either OGT or OGA. Interestingly, they observed that in wild-type animals, *O*-GlcNAc marks on promoters were surprisingly very similar in fed and starved conditions, but responded aberrantly to nutrient flux when *O*-GlcNAc cycling was blocked by OGA knock out. They suggested that in wild-type animals, the dynamic cycling of *O*-GlcNAc is required to maintain buffered levels of *O*-GlcNAcylation at gene promoters, reminiscent of the optimal intracellular *O*-GlcNAcylation level proposed by Ong et al..

*O*-GlcNAcylation is nearly universal in the living kingdom. This was further demonstrated in parasites by two original studies, in which *O*-GlcNAcylated proteins were identified in *Toxoplasma gondii* (Aquino-Gil et al.) and *Trypanosoma cruzi* (Torres-Gutiérrez et al.). Identification of *O*-GlcNAc on proteins involved in invasion, such as rhoptries in *T. gondii*, or in microtubules formation in *T. cruzi*, may suggest new therapeutic strategies against infections by these parasites.

A little on the fringes of what could be considered today as the classical *O*-GlcNAcylation pathway, that is modification of nucleocytoplasmic proteins, Nagnan-Le Meillour et al. focused on odorant-binding proteins (OBP) secreted in the nasal mucus. By a set of different approaches including mass spectrometry, they managed to identify several sites of *O*-GlcNAcylation and phosphorylation on OBP. Interestingly, they showed that phosphorylated isoforms of OBP only slightly modify interaction with lipid ligands, whereas O-GlcNAcylation of OBP favors binding. These data reveal a new regulatory mechanism by which PTMs and specifically extracellular *O*-GlcNAcylation, managed by EOGT (the *endoplasmic reticulum*-resident form of OGT), can modulate recognition of odorant molecules by OBP, enlarging the panel of odors discrimination.

Finally, an interesting technical contribution to this Research Topic is dedicated to the development of a novel *O*-GlcNAcomics workflow based on GalNAz labeling of cells and quantitative proteomics analysis Cox et al. As a proof of concept Cox et al. identified *O*-GlcNAcylation of COP1γ (a component of COPI). They pinpointed several *O*-GlcNAc sites on COP1γ and proposed that *O*-GlcNAc is a regulator of mammalian vesicle trafficking within the Golgi apparatus and from the Golgi to the *endoplasmic reticulum*.

Looking back through the series of reviews and original articles gathered here, we feel that this Research Topic has been an opportunity for several young researchers to present their first work on this particularly exciting modification. As such, it may reflects the beginning of a new era of investigation on *O*-GlcNAcylation. Because of its wide distribution in the living world and the numerous biological processes it controls, there is no doubt that in the near future other new teams will get on board for the study of *O-*GlcNAc.

## Author Contributions

All authors listed have made a substantial, direct and intellectual contribution to the work, and approved it for publication.

### Conflict of Interest

The authors declare that the research was conducted in the absence of any commercial or financial relationships that could be construed as a potential conflict of interest.
